# Cyber-bullying among university students: Concurrent relations to belief in a just world and to empathy

**DOI:** 10.1007/s12144-022-03239-z

**Published:** 2022-06-01

**Authors:** Matthias Donat, Anna Willisch, Anett Wolgast

**Affiliations:** 1 Department of Educational Psychology, Martin Luther University of Halle-Wittenberg, Franckeplatz 1, D-06099 Halle/Saale, Germany; 2 Department of Psychology, University of Applied Sciences, Hanover, Germany

**Keywords:** Cyber-bullying, Belief in a just world, Lecturer justice, Fellow student justice, Empathy, Higher education

## Abstract

The aim of this study was to investigate concurrent relations of belief in a just world (BJW), justice experiences, and empathy to cyber-bullying perpetration and victimization. Our theoretical framework contained a distinction between personal and general BJW and between affective and cognitive empathy. Due to theory and recent research, we expected students’ BJW, lecturer and fellow student justice, and empathy to relate negatively to cyber-bullying perpetration. Furthermore, BJW and academic justice experiences are also expected to negatively relate to cyber-bullying victimization. In order to test our hypotheses, we conducted a cross-sectional questionnaire study with *N* = 663 German university students (*M*_age_ = 22.6, *SD*_age_ = 3.5; 68% female). Gender, internet use, and social desirability were considered as control variables. A structural equation model showed that students’ personal BJW, academic justice experiences, and both empathy dimensions but not general BJW negatively related to cyber-bullying perpetration. Additionally, students’ personal BJW and academic justice experiences but not general BJW or empathy were associated with cyber-bullying victimization. Male students and those with a low social desirability were more likely to report cyber-bullying perpetration and victimization. Altogether, not only university students’ personal BJW and empathy but also their academic justice experiences related to cyber-bullying perpetration or victimization. Thus, researchers should develop strategies aiming to prevent or reduce cyber-bullying. Those should simultaneously foster students’ personal BJW and empathy as well as an academic environment characterized by just behavior of lecturers and students.

**Supplementary Information:**

The online version contains supplementary material available at 10.1007/s12144-022-03239-z.

## Introduction

Cyber-bullying represents a special form of bullying and is quite a new phenomenon especially in higher education and therefore underrepresented in empirical research. Additionally, studies have shown that cyber-bullying among young people seems to be most prevalent in higher education (Kokkinos et al., 2014) and that cyber-bullying experiences were relatively stable even after young people’s transition from school to university (Larrañaga et al., 2016). Thus, a major interest of research on cyber-bullying among university students is to identify individual factors that might explain this phenomenon.

Cyber-bullying can be understood as unjust and deviant behavior because cyber-bullying perpetrators violate social norms and interpersonal rules. They also infringe on the personal rights of victimized people, who undeservedly suffer from the serious outcomes of cyber-bullying threatening their mental health and educational success. Consequently, cyber-bullying might be investigated from a justice-psychological perspective and be explained by inter-individual differences in the belief in a just world (BJW; Lerner, 1980). In recent studies (Donat et al., 2020; Donat et al., 2018a, b), researchers have already shown negative relations between *school* students’ BJW and their self-reported offline as well as cyber-bullying behavior or victimization. Given the prevalence of cyber-bullying in higher education, it seems necessary to investigate a just-world approach in relation to cyber-bullying also among *university* students because it would provide helpful indications regarding generalizable justice-psychological processes in young people.

Moreover, the students’ empathy seems to play an important role as well in explaining their cyber-bullying behavior because it is considered a fundamental resource enhancing social interactions and prosocial behavior, but also inhibiting antisocial acts (Del Rey et al., 2016; Jolliffe & Farrington, 2004). Accordingly, studies have shown negative relations between school students’ empathy and self-reported offline and cyber-bullying (Del Rey et al., 2016; Donat et al., 2020). In the current study, we aimed to investigate the relations of BJW and empathy with cyber-bullying concurrently in a sample of university students.

Belief in a just world and empathy are so-called proximal attributes of Bierhoff’s (2002, 2010) model of the prosocial personality, which is defined as “an enduring tendency to think about the welfare and rights of other people, to feel concern and empathy for them, and to act in a way that benefits them” (Penner & Finkelstein, 1998, p. 526). In contrast, people with a strong prosocial personality are unlikely to promote antisocial behavior such as aggression or cyber-bullying. This model also includes social factors (distal attributes) that belong to the community people interact with. Thus, in this study we also included students’ experiences in their academic environment, that is, their experiences of just behavior of their lecturers and fellow students, as another factor that might explain cyber-bullying experiences.

### Cyber-Bullying

In his critical review, Tokunaga (2010, p. 278) integrated a number of definitions of cyber-bullying and defined it as “any behavior performed through electronic or digital media by individuals or groups that repeatedly communicates hostile or aggressive messages intended to inflict harm or discomfort on others.” Meanwhile, there are lots of digital devices and media used to cyber-bully others. According to Watts et al. (2017), emails, texting, and instant messaging seem to be the most popular formats. Additionally, social networking sites such as Tik Tok, Twitter, or Instagram seem to be of increasing importance for example because of the possibility of easily sharing videos and photos and due to the masses of people who may witness and/or participate in cyber-bullying incidents.

Definitions of cyber-bullying seem to be partly based on definitional criteria of offline bullying. For example, similar to offline bullying (e.g., Olweus, 1993), cyber-bullying is characterized by *repetition* of negative acts and *intentionality*. Yet the repetitive nature of cyber-bullying takes a unique form because digital messages, videos, memes, or images can easily be distributed, stored offline, and viewed at a later time. Furthermore, these materials often persist online and are hard to delete, leading to repeated effects once they have been uploaded (e.g., Brody & Vangelisti, 2017; Menesini & Nocentini, 2009; Slonje & Smith, 2008).

However, there are also criteria which are specific to cyber-bullying definitions, for example, *anonymity* (Foody et al., 2015; Kowalski et al., 2014; Tokunaga, 2010), which means that cyber-bullying perpetrators usually experience themselves as anonymous (Kowalski et al., 2014). Online environments enable them to easily evade punishment as they mostly remain unidentified (Foody et al., 2015; Sticca & Perren, 2013). In anonymous situations, people did and said things they would not do or say in face-to-face interactions (Diener, 1980; Kowalski et al., 2014). These results of anonymity are subsumed under the so-called online disinhibition effect (e.g., Suler, 2004). Furthermore, this anonymity seems to be especially frightening to cyber-victimized people and makes them helpless and powerless; thus, cyber-bullying is perceived as being more severe than offline-bullying (for a review, e.g., Sticca & Perren, 2013).

In addition, the *time*, *location*, and *context* of cyber-bullying episodes fluctuate widely (Brody & Vangelisti, 2017; Kowalski et al., 2014; Tokunaga, 2010). Offline bullying takes place most frequently during class time and often stops once victims have returned home (Nansel et al., 2001), but cyber-bullying can be perpetrated any time and any place during the day or night and is thus independent of the academic institution or context (Foody et al., 2015; Kowalski et al., 2014). Due to the endurance of cyber-bullying, such incidents have even greater negative effects than offline bullying (e.g., Watts et al., 2017).

Differences between victim and perpetrator in *physical strength* are less obvious in online than offline settings. Consequently, the *power imbalance* that usually characterizes offline bullying seems to be less meaningful online (Brody & Vangelisti, 2017; Menesini & Nocentini, 2009). However, due to the anonymity of cyber-bullying perpetrators, their superior digital skills, and the public nature of cyber-bullying acts, the power imbalance might at least take a different form in cyber-bullying than in offline-bullying incidents (e.g., Betts, 2016). Besides, the potential *audience* in cyber-bullying incidents is larger than offline: hundreds or even thousands of people can watch injurious posts online, but only a small number of people are likely to witness offline bullying incidents (Kowalski et al., 2014).

Prevalence estimates of cyber-bullying at school differ widely across studies and countries and range from 10 to 40% (Buelga et al., 2017; Foody et al., 2015; Hinduja & Patchin, 2008; Kowalski et al., 2014; Tokunaga, 2010). Studies also showed that cyber-bullying is highly consistent after transition from school to higher education (e.g., Larrañaga et al., 2016; Watts et al., 2017), that is, students who perpetrated cyber-bullying or were cyber-bullied at school are very likely to experience such situations also in higher education. Additionally, cyber-bullying among young people seems to be even most prevalent in higher education (e.g., Kokkinos et al., 2014). This might be due to the fact that young adults use the internet and social media most intensively and seem to disclose large parts of their private lives in a relatively uncontrolled manner. More precisely, prevalence rates of cyber-bullying in higher education range from 4 to 60% who reported that they have cyber-bullied others and from 19 to 72% who reported that they have been cyber-bullied (MacDonald & Roberts-Pittman, 2010; Musharraf & Anis-ul-Haque, 2018; Xiao & Wong, 2013; Zalaquett & Chatters, 2014). In a recent international review, Larrañaga et al. (2016) reported prevalence rates between 2.2 and 9% for cyber-bullying perpetrators and rates between 8 and 81% for cyber-bullying victims. However, authors such as Larrañaga et al. (2016) emphasize that prevalence rates are hard to compare due to several factors that differ across studies, especially criteria to identify cyber-bullying perpetrators or victims, different measurement scales, and the time frame in which cyber-bullying incidents have occurred.

Recent literature reviews emphasize that consequences of cyber-bullying experiences among college students are at least as serious as those of cyber- and offline bullying among school students. Among others, frequent cyber-bullying experiences among students in higher education are associated with poor social functioning, increased psychological stress, negative feelings (e.g., sadness, fear), mental disorders (e.g., depression, social anxiety, insomnia), suicide and/or suicidal ideation, increased substance use (e.g., alcohol), decreased self-esteem and academic performance, avoidance or abandonment of studies (e.g., Larrañaga et al., 2016; Lei et al., 2020; Mukherjee & Hussain, 2020; Watts et al., 2017; Zalaquett & Chatters, 2014). Altogether, cyber bullying thus threatens students’ mental health and study success.

In past research, offline bullying was more likely to occur among boys than girls (Scheithauer et al., 2006), but gender differences in cyber-bullying were less clear (e.g., MacDonald & Roberts-Pittman, 2010). More recent studies on students in higher education showed that men cyber-bullied others more often than women (e.g., see Kokkinos et al., 2014, for a review; Zalaquett & Chatters, 2014) whereas prevalence rates of cyber-bullying victimization indicate a higher risk for women (e.g., Kokkinos et al., 2014; Larrañaga et al., 2016).

Students who more frequently used the internet and spent more time online were more likely to become a cyber-bullying victim or perpetrator (e.g., see Kokkinos et al., 2014, for a review). Thus, frequent internet use seems to be a risk factor for cyber-bullying. However, there are also studies that showed no significant relation between intense internet use and cyber-bullying among university students (e.g., Zalaquett & Chatters, 2014).

People with a strong tendency to give favorable self-descriptions usually have a high level of social desirability or socially desirable responding (Paulhus, 2002). Those people typically avoid straying from desired social norms and act in a socially adapted way. This tendency seems to be particularly crucial when people are invited to uncover sensitive information, for example self-report criminal behavior or illicit drug use (see Tourangeau & Yan, 2007, for a review). A strong social desirability is considered as a type of response bias and is thus likely to relate to people’s reports about deviant behavior or experiences of suffering. More specifically, individuals with a strong social desirability are less willing to honestly self-report perpetration or victimization (e.g., Bell & Naugle, 2007), which might also be true when university students report their experiences of cyber-bullying.

### Cyber-Bullying and Belief in a Just World

Cyber-bullying behavior cannot only be considered as hostile or aggressive but also as deviant and unjust. From the perspective of justice psychology, such behavior breaches social rules and norms, violates the personal rights of victimized people and simultaneously principles and criteria of justice (e.g., interpersonal justice). Additionally, innocent cyber-bullying victims suffer from the serious consequences of online attacks which they do not deserve; in contrast, cyber-bullying perpetrators usually remain undetected and escape punishment although they deserve it. Consequently, cyber-bullying might be associated with individual differences in people’s conviction that they live in a world in which bad things happen to bad people and good things happen to good people.

Such a conviction is the BJW, originating from Lerner’s just-world hypothesis (e.g., Lerner, 1980; Lerner & Simmons, 1966; see also Hafer & Sutton, 2016). This hypothesis contains the idea that people need to believe in a world where all deserve what they get and get what they deserve. Then, a world that functions this way is just. The BJW enables people to interact with their social environment as if it were predictable, stable, and orderly. Thus, this belief serves significant adaptive functions. Three of these functions (e.g., Dalbert, 2001; for a review, see also Dalbert & Donat, 2015) have been intensively investigated in recent research (Kiral Ucar et al., 2019).

First, the *motive function* obligates people to act justly in order to support the justice of the world. Here, BJW indicates a personal contract (Lerner, 1980) in which people strive to behave justly towards others and such behavior, they believe, will be rewarded in the future (Dalbert, 2001; Lerner, 1977). This contains, for example, people’s motivation to achieve personal goals by just means and to avoid unjust behavior which would mean a breach of the personal contract (for a review, see Dalbert & Donat, 2015). This is also in line with the above-mentioned model of the prosocial personality (Bierhoff, 2002, 2010) that includes BJW as a proximal attribute. In this sense, BJW was associated with less rule-breaking behavior, academic dishonesty, and delinquent behavior in adolescents and young adults (for a review, see Dalbert & Donat, 2015; Donat et al., 2014; Münscher et al., 2020). By extension, school students with a strong BJW were more likely to avoid offline and cyber-bullying behavior than others (Correia & Dalbert, 2008; Donat et al., 2012, 2018a, 2020).

Second, the *assimilation function* helps just-world believers preserve BJW when confronted with injustice which threatens BJW. This means that people restore justice psychologically when they are unable or convinced of not being able to do so by their own actions, for example by minimizing or denying the injustice (Lipkus & Siegler, 1993), avoiding self-focused rumination (Dalbert, 1997), or forgiving (Strelan, 2007). As a consequence, people with a strong BJW felt more justly treated by others than those with a weak BJW (for a review, see Dalbert & Donat, 2015). In higher education, students’ BJW can thus be expected to relate positively to their experiences of being justly treated by their lecturers and fellow students (Münscher et al., 2020)—a relation that has similarly been identified among students at school (e.g., Correia & Dalbert, 2007; Dalbert & Stöber, 2005). In accordance with research on the connection between school students’ BJW and offline as well as cyber-bullying victimization (e.g., Correia & Dalbert, 2008; Donat et al., 2018b, 2020), BJW can function as a psychological resource even for victimized students. This might be particularly crucial because victimized students are often helpless and fail to defend themselves (e.g., Wachs et al., 2016). The assimilation function can help these students cope with unjust experiences of victimization, for example through cognitive reframing. This means that students with a strong BJW are likely to deny or at least minimize such experiences or forgive the perpetrators.

Third, the *trust function* enables individuals to have trust in the justice of their fate and in other people. As adaptive consequences, this trust provides people with the assurance to be able to invest in long-term goals and to be justly treated and rewarded (e.g., Hafer, 2000; Sutton & Winnard, 2007). In the academic context, students with a strong BJW are likely to show an increased trust in their lecturers’ or fellow students’ just treatment or judgment (for a review, see Dalbert & Donat, 2015; Münscher et al., 2020).

Just-world research indicates the necessity to differentiate two dimensions of BJW which might have varying psychological functioning: the personal BJW and the general BJW (e.g., Dalbert, 1999). *Personal BJW* is focused on people’s direct or near social environment and represents their conviction that they are usually treated justly. In studies, it was a better indicator of the justice motive and more strongly related to subjective well-being (for a review, see Dalbert & Donat, 2015; Hafer & Sutton, 2016). In contrast, *general BJW* is people’s conviction that the world as a whole is a just place (Dalbert et al., 1987) and seems to be a better predictor of harsh social attitudes (Dalbert & Donat, 2015; Hafer & Sutton, 2016). However, researchers repeatedly show that both BJW dimensions are positively correlated and even argue that they form a common latent factor ‘BJW’ which was particularly associated with well-being, but each dimension may also contribute uniquely to other adaptive psychological outcomes (e.g., Bartholomaeus & Strelan, 2019; Hafer et al., 2020). Consequently, we considered both personal and general BJW in our study.

### Cyber-Bullying and Justice Experiences

Bierhoff’s (2002, 2010) model of the prosocial personality also includes distal factors such as people’s interactions and experiences with their social community. Accordingly, recent research has shown that adaptive outcomes among students such as appropriate social behavior were not only explained by justice convictions but also justice experiences in important social environments (e.g., school). Thus, researchers have started to focus more on the importance of such experiences in the school and academic context. Among students in higher education, two groups of people are especially meaningful sources of justice experiences, namely lecturers and fellow students. Thus, the students’ individual justice experiences with these groups were of particular interest to our study. In line with definitions of teacher (Dalbert & Stöber, 2006) and classmate justice (Correia & Dalbert, 2007), we define lecturer/fellow student justice as the students’ individually and subjectively experienced justice of their lecturers’/fellow students’ behavior toward them (“they-to-me approach”; Peter & Dalbert, 2010).

In accordance with the adaptive functions of BJW, students’ BJW is positively related to lecturer and fellow student justice, which was supported in recent studies in the school and academic context (e.g., Donat et al., 2020; Münscher et al., 2020). Furthermore, justice experiences have been shown to be negatively associated with rule-breaking and deviant behavior such as academic cheating or offline and cyber-bullying at school (e.g., Donat et al., 2020; Münscher et al., 2020). Additionally and in accordance with the Group-Value Theory (Lind & Tyler, 1988), just treatment by lecturers or fellow students indicates that students are valued members of their social group within the academic context. This appreciation may foster feelings of social inclusion and belonging (e.g., Umlauft & Dalbert, 2017) which in turn motivate students to observe and accept norm and rules in the academic context and even beyond (e.g., Sanches et al., 2012; Thomas & Mucherah, 2018) and consequently avoid cyber-bullying perpetration. There are also some clues in recent studies that feelings of esteem, belonging, and social inclusion—sustained by justice experiences—may promote students’ well-being and make victimization by peers unlikely (e.g., Donat et al., 2020; Münscher et al., 2020).

### Cyber-Bullying and Empathy

In line with Bierhoff’s (2002, 2010) model, empathy as another proximal attribute of the prosocial personality represents an important resource that enhances social interactions and prosocial behavior but also inhibits antisocial acts (Del Rey et al., 2016; Jolliffe & Farrington, 2004). After decades of theorizing and research, empathy to date is conceptualized within a multi-dimensional framework which contains an emotional or affective disposition and a cognitive disposition (Davis, 1980; Wolgast et al., 2020). The affective dimension—often called emotional concern (e.g., Davis, 1980) or just *affective empathy*—is defined as an individual’s cross-contextual tendency to emotionally share other people’s feelings in their circumstances. The cognitive dimension—perspective taking or *cognitive empathy* (e.g., Davis, 1980)—is defined as an individual’s cross-contextual tendency to imagine and understand other people’s perspectives, mental circumstances, and feelings but without sharing their emotions.

In general, empathy is considered a fundamental resource of people, which facilitates social interactions and interpersonal communication (Del Rey et al., 2016) and is essential for prosocial acts (Jolliffe & Farrington, 2004). As people with a strong empathic tendency are able to understand and share victims’ suffering, they avoid behaving violently themselves. This is supported by many studies in which empathy was positively associated with helping and defending and seems to inhibit aggression among people of different ages (e.g., Correia & Dalbert, 2008; Espelage et al., 2018; Fredrick et al., 2020; Miller & Eisenberg, 1988). Studies on the relation between empathy and school-related forms of aggression such as offline and cyber-bullying consistently showed negative associations (e.g., Brewer & Kerslake, 2015; Del Rey et al., 2016; van Noorden et al., 2015; Zych et al., 2019). These findings have been replicated among university students (e.g., Kokkinos et al., 2014), with affective empathy more strongly relating to perpetration than cognitive empathy. Results regarding the relations between affective or cognitive empathy and victimization were mixed and less clear (e.g., Brewer & Kerslake, 2015; Del Rey et al., 2016; Kokkinos et al., 2014), ranging from low negative to insignificant effects.

### Current Study

The major aim of our study was a first-time investigation of concurrent relations between justice beliefs and experiences as well as empathy among university students on the one hand and their cyber-bullying perpetration and victimization on the other hand. This also enables us to replicate previous findings among school students and generalize them to the academic context. More specifically, we tested the following hypotheses: The stronger university students’ BJW (1), the less likely they are to cyber-bully others, (2) the less likely they are to report being victims of cyber-bullying by others, and (3) the more likely they are to judge their lecturers’ and fellow students’ behavior toward them personally as just. The more students evaluate their lecturers’ or fellow students’ behavior as just, (4) the less likely they are to cyber-bully others, and (5) the less likely they are to report experiences of cyber-bullying victimization. (6) The stronger students’ empathy, the less cyber-bullying perpetration they report. Moreover, these relations are significant when controlled for confounding effects of gender, internet use, and social desirability.

## Method

### Sample and Procedure

The participants in our study were German university students who were in two samples with *n*_1_ = 363 (*M*_age_ = 22.6, *SD*_age_ = 3.4; 65.6% female) and *n*_2_ = 252 (*M*_age_ = 22.6, *SD*_age_ = 3.7; 70.6% female); two students of sample 1 and 2 each did not indicate their gender; 14 students of sample 1 and one student of sample 2 did not indicate their age.

University students were invited to complete a questionnaire assessing justice and experiences at university. It was stressed that participation was anonymous and voluntary. Participants of the first subsample took part about 2–3 months before the SARS-CoV-2 pandemic started and completed a paper-and-pencil questionnaire; participants of the second subsample took part about 2–3 months after the beginning of the SARS-CoV-2 pandemic via an online questionnaire.

At the end of the questionnaire, the participants indicated whether or not they possessed a mobile phone/smart phone, a computer, and/or internet access (via phone or computer). They also indicated how many hours per day they spent on the internet on average. We excluded students who (1) indicated that they possessed none of these devices because the usage of at least one such digital or electronic medium is a necessary criterion of cyber-bullying, (2) made implausible statements, or (3) did not complete the questionnaire. Due to these exclusion criteria, we excluded a total of *n* = 48 cases from the study, which originally consisted of 663 university students.

### Measures

*Personal BJW* was measured using the one-dimensional Personal Belief in a Just World Scale (Dalbert, 1999), with seven items that capture the belief that, overall, events in a person’s life are just; sample item: “Overall, events in my life are just”; (Cronbach’s α_1_ = .83, α_2_ = .84; McDonald’s ω_1_ = ω_2_ = .88; α ranged in other studies between α = .82 and α = .87, Dalbert, 1999).

*General BJW* was measured using the one-dimensional General Belief in a Just World Scale (Dalbert et al., 1987), with six items capturing the belief that the world as a whole is a just place; sample item: “I think basically the world is a just place”; (α_1_ = α_2_ = .77; ω_1_ = .82, ω_2_ = .84; α = .82 in other studies, Dalbert et al., 1987).

We assessed the affective empathy dimension *emotional concern* using four items of Davis’s (1980) Interpersonal Reactivity Index (IRI; German version: *C.* *Paulus*, 2009) capturing people’s vicarious or other-oriented emotional responses such as compassion and concern toward perceived need of others; sample item: “I am often quite touched by things that I see happen” (α_1_ = .65, α_2_ = .71; ω_1_ = .68, ω_2_ = .74; α ranged in other studies between α = .71 and α = .77, *C.* *Paulus*, 2009).

The cognitive empathy dimension *perspective taking* was measured using four items of Davis’s (1980) Interpersonal Reactivity Index (IRI; German version: *C.* *Paulus*, 2009) that capture people’s tendency to anticipate others’ reaction and behavior; sample item: “I sometimes try to understand my friends better by imagining how things look from their perspective” (α_1_ = α_2_ = .73; ω_1_ = .88, ω_2_ = .80; α ranged in other studies between α = .56 and α = .77, *C.* *Paulus*, 2009).

We assessed *lecturer justice* using an adapted version of the one-dimensional Teacher Justice Scale (Dalbert & Stöber, 2002) with 10 items which were designed to capture university students’ experience of the justice of their lecturers’ behavior toward them personally; sample item: “My lecturers generally treat me justly” (α_1_ = .86, α_2_ = .87; ω_1_ = ω_2_ = .90; α ranged in other studies between α = .74 and α = .90, Dalbert & Stöber, 2002).

*Fellow student justice* was investigated using an adapted version of the one-dimensional Classmate Justice Scale (Correia & Dalbert, 2007) comprising six items capturing university students’ experience of the justice of their fellow students’ behavior toward them personally; sample item: “My fellow students generally treat me justly” (α_1_ = .86, α_2_ = .88; ω_1_ = .91, ω_2_ = .95; α ranged in other studies between α = .82 and α = .97, Correia & Dalbert, 2007).

To measure *social desirability,* we used 10 items from the one-dimensional Social Desirability Scale-17 (Stöber, 2001); sample item: “I always accept others’ opinions, even when they don’t agree with my own” (α_1_ = .71, α_2_ = .75; ω_1_ = .75, ω_2_ = .79; α ranged in other studies between α = .74 and α = .75, Stöber, 2001).

Participants indicated their responses to all of the above-mentioned scales on a six-point scale ranging from 1 (*totally disagree*) to 6 (*totally agree*). In an open-ended item, participants indicated the duration of their average daily internet use: “How many hours a day on average do you spend on the internet?” (*M*_*1*_ = 3.53, *SD*_*1*_ = 1.91; *M*_*2*_ = 4.57, *SD*_*2*_ = 2.42).

We assessed self-reported *cyber-bullying perpetration* using the 11-item one-dimensional perpetrator subscale from the European Cyberbullying Intervention Project Questionnaire (Del Rey et al., 2015; German version: Schultze-Krumbholz et al., 2011). It was designed to capture students’ behavior intended to bully others using digital devices; sample item: “I said nasty things about someone to other people either online or through text messages” (α_1_ = .87, α_2_ = .96; ω_1_ = .91, ω_2_ = .98). The items were introduced by the sentences: “In the past 2 months, have you engaged in any of the following behaviors online or via mobile phone? For each behavior, please indicate which answer applies to you!” In another study, α was .93 (Del Rey et al., 2015).

We measured self-reported *cyber-bullying victimization* using the 11-item one-dimensional victim subscale from the European Cyberbullying Intervention Project Questionnaire (Del Rey et al., 2015; German version: Schultze-Krumbholz et al., 2011). It was designed to capture students’ victimization experiences when using digital devices; sample item: “Someone hacked into my account and pretended to be me (e.g., through instant messaging or social networking accounts)” (α_1_ = .83, α_2_ = .93; ω_1_ = .88, ω_2_ = .95). The items were introduced by the sentences: “In the past 2 months, have you experienced any of the following behaviors online or via mobile phone? For each behavior, please indicate which answer applies to you!” In another study, α was .97 (Del Rey et al., 2015).

Responses to the cyber-bullying items were indicated on a five-point scale ranging from 1 (*no*) to 5 (*yes, more than once a week*). We formed scale scores by averaging the responses across items, reverse coding negative items as necessary. Missing rates ranged from 0 to 3% over all measures. Cronbach’s α and McDonald’s ω were calculated in the R environment by the package *psych* (R Development Core Team, 2009; Revelle, 2019). Table 1 presents means, standard deviations, skewness, and kurtosis of the study scales.

**Table 1:** Mean, standard deviation, skewness, and kurtosis of responses on study scales

	*M*	*SD*	Skewness	Kurtosis
Personal BJW	4.49	0.69	−0.68	0.75
General BJW	3.08	0.87	0.02	−0.33
Affective empathy	4.54	0.80	−0.49	0.06
Cognitive empathy	4.55	0.78	−0.36	−0.20
Lecturer justice	4.92	0.69	−0.88	0.85
Fellow student justice	5.05	0.72	−1.23	2.13
Cyber-bullying perpetration	1.11	0.26	6.29	50.08
Cyber-bullying victimization	1.16	0.32	4.47	28.23

*BJW* belief in a just world. Cyber-bullying perpetration and victimization ranged between 1 and 5, all other variables between 1 and 6, with higher values indicating a stronger endorsement of the constructs

### Statistical Analyses

In accordance with our research question, which was about the extent to which BJW, justice experiences, and empathy concurrently relate to cyber-bullying perpetration and victimization, we evaluated the model fit of a confirmatory factor analysis (CFA) including nine latent factors. For example, the 11 items concerning cyber-bullying victimization (indicators) were used to measure the unobservable construct (1) *cyber-bullying victimization* (latent factor) while accounting for measurement error. In the same way, the items concerning personal BJW, general BJW, affective empathy, cognitive empathy, lecturer justice, fellow student justice, cyber-bullying perpetration, and social desirability (indicators) were used to measure the unobservable constructs (2) *personal BJW,* (3) *general BJW,* (4) *affective empathy,* (5) *cognitive empathy,* (6) *lecturer justice,* (7) *fellow student justice,* (8) *cyber-bullying perpetration*, and (9) *social desirability*.

**Fig. 1:**
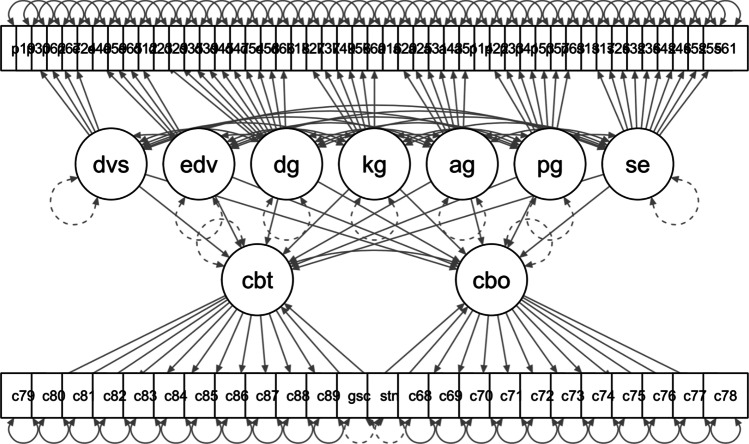
Tested structural equation model. *Note*. SEM including nine latent factors: dvs = cognitive empathy, edv = affective empathy, dg = lecturer justice, kg = fellow student justice, ag = general belief in a just world, pg = personal belief in a just world, se = social desirability, cbt = cyber-bullying perpetration, cbo = cyber-bullying victimization, latent autocorrelations, and the latent factor’s indicators each; two manifest factors: gsc = gender, str = internet use; and residuals

Then, the CFA model was expanded to a latent structural equation model (SEM). The advantage of SEM is that it accounts for the measurement error of the nine latent factors and also allows the modelling of concurrent relations (latent correlations) between all latent factors (in contrast to the analysis of manifest bivariate correlations). Thus, the SEM was used to measure the relations between the nine latent factors *personal BJW, general BJW, affective empathy, cognitive empathy, lecturer justice, fellow student justice, cyber-bullying victimization, cyber-bullying perpetration*, and *social desirability* while taking into account the control variables. This means that additional regression paths were included from each of the manifest control variables *gender* (female, male) and *internet use* to each of the nine latent factors. The control variable social desirability was also included as a latent factor to account for measurement error. The CFA and SEM were constructed with the R package *lavaan* (R Development Core Team, 2009; Rosseel et al., 2018). All variables were z-standardized and means- and variance-adjusted weighted least squares estimation was applied (WLSMV estimation; Rosseel et al., 2018). Figure 1 shows the tested model.

## Results

The CFA suggested a good fit between the model including nine factors and the data: χ^2^(2241) = 2862.54; comparative fit index (CFI) = .97; Tucker-Lewis index (TLI) = .96; normed fit index (NFI) = .96; root mean square error of approximation (RMSEA) = .02, 95% CI [.022, .024]; standardized root mean square residual (SRMR) = .07. Multi-group analyses using this CFA model with regard to both subsamples (two groups) suggested measurement invariance for item loadings (ΔCFI = .006, ΔRMSEA < .001), item intercepts (ΔCFI = .001, ΔRMSEA < .001), and item means (ΔCFI = .006, ΔRMSEA < .001). This scalar measurement invariance allowed us to analyze one data set including all *n* = 615 cases in our further analyses.

The SEM indicated a good model fit: χ^2^(2361.00) = 3000.01; CFI = .97; TLI = .96; NFI = .96; SRMR = .07; RMSEA = .022, 95% CI [.019; .024]. The analysis (see Table 2) revealed that personal but not general BJW negatively related to cyber-bullying perpetration (*Corr*_(lat)_ = −.19, *p* < .01) and victimization (*Corr*_(lat)_ = −.25, *p* < .01). The more students endorsed the personal BJW, the less likely they were to self-report cyber-bullying perpetration and victimization. The results also showed that students who felt justly treated by their lecturers or fellow students were less likely to self-report cyber-bullying perpetration (*Corr*_(lat)_ = −.18, *p* < .01; *Corr*_(lat)_ = −.12, *p* = .03) and victimization (*Corr*_(lat)_ = −.26, *p* < .01; *Corr*_(lat)_ = −.23, *p* < .01) than students who did not. These justice experiences were positively related to personal but not general BJW (*Corr*_(lat)_ = .53, *p* < .01; *Corr*_(lat)_ = .54, *p* < .01). Furthermore, affective (*Corr*_(lat)_ = −.18, *p* < .01) and cognitive empathy (*Corr*_(lat)_ = −.20, *p* < .01) also negatively related to cyber-bullying perpetration but not victimization. Students who had a strong tendency to give socially desirable responses (*Corr*_(lat)_ = −.28, *p* < .01; *Corr*_(lat)_ = −.21, *p* < .01) and female students (*Corr*_(lat)_ = .23, *p* < .01) (*Corr*_(lat)_ = .22, *p* < .01) were less likely to self-report cyber-bullying perpetration and victimization than their counterparts. Internet use was unrelated to cyber-bullying perpetration and victimization. Additional results are provided in the supplemental material.

**Table 2:** Latent correlations and regression weights from a latent structural equation model (*n* = 615 German University Students)

Variables	*Corr*_(lat)_	*SE*	*z*	*p*	CI_95%_	
Cyber-bullying perpetration ~~						
Personal BJW	−.19	0.05	−4.23	< .01	−.28,	−.10
General BJW	−.06	0.06	−1.06	.29	−.17,	.05
Affective empathy	−.18	0.06	−3.21	< .01	−.29,	−.07
Cognitive empathy	−.20	0.04	−4.43	< .01	−.28,	−.11
Lecturer justice	−.18	0.06	−3.05	< .01	−.29,	−.06
Fellow student justice	−.12	0.06	−2.17	.03	−.23,	−.01
Social desirability	−.28	0.06	−4.69	< .01	−.40,	−.17
Cyber-bullying victimization ~~						
Personal BJW	−.25	0.05	−4.96	< .01	−.35,	−.15
General BJW	< .01	0.05	0.03	.98	−.10,	.10
Affective empathy	−.11	0.06	−1.79	.07	−.22,	.01
Cognitive empathy	−.11	0.06	−1.77	.08	−.22,	.01
Lecturer justice	−.26	0.06	−4.72	< .01	−.37,	−.15
Fellow student justice	−.23	0.05	−4.26	< .01	−.33,	−.12
Social desirability	−.21	0.08	−2.80	.01	−.36,	−.06
Personal BJW ~ ~						
Lecturer justice	.53	0.04	12.22	< .01	.45	.62
Fellow student justice	.54	0.05	12.03	< .01	.45	.63
General BJW ~ ~						
Lecturer justice	< .01	0.05	−0.02	.98	−.10	.10
Fellow student justice	−.02	0.06	−0.40	.69	−.13	.09
Variables	β	*SE*	*z*	*p*	CI_95%_	
Cyber-bullying perpetration ~						
Gender	.23	0.05	4.50	< .01	.13	.33
Internet use	.05	0.03	1.42	.16	−.02	.12
Cyber-bullying victimization ~						
Gender	.22	0.06	3.54	< .01	.10	.34
Internet use	.05	0.04	1.34	.18	−.02	.12

*Corr*_(lat)_ latent correlation. *BJW* belief in a just world. Cyber-bullying perpetration and victimization ranged between 1 and 5, all other variables between 1 and 6, with higher values indicating a stronger endorsement of the constructs. For gender, 1 = female, 2 = male. Internet use: average hours spent daily on the internet

## Discussion

In our study, we investigated the concurrent relations between university students’ cyber-bullying perpetration as well as victimization and their BJW, empathy, and subjective experiences of lecturers’ and fellow students’ justice, while we statistically controlled for gender, internet use, and social desirability.

In accordance with our first hypothesis, personal but not general BJW negatively related to cyber-bullying perpetration. This result is consistent with the idea that personal BJW indicates a justice motive and contains a personal contract (e.g., Lerner, 1980). Thus, students with a strong personal BJW are obligated to behave justly and avoid unjust behavior such as cyber-bullying others. This finding is further in line with results from recent studies on offline and cyber-bullying in adolescents (Donat et al., 2018a, 2020) and supports the expectation of personal BJW being negatively connected with university students’ unjust behavior. One such behavior is cyber-bullying that clearly violates rules of justice and strong just-world believers’ personal contract. Together with findings of studies on the relation of personal BJW to other kinds of rule-breaking behavior or delinquent intensions (for a review, Dalbert & Donat, 2015; Donat et al., 2014), we interpret our results even in a more general way, meaning that personal BJW helps people of different ages avoid a variety of unjust behaviors.

Additionally, our findings support the assimilation and trust function of BJW (Dalbert, 2001; Lerner, 1980) regarding experiences of cyber-bullying victimization. Personal BJW may enable cyber-victimized university students to cope with such experiences, negative emotions, and adverse life events in general. In line with this and our second hypothesis, results showed students with a strong personal BJW to be less likely to report being cyber-bullied by others. Cognitive reframing for example, which is part of BJW’s assimilation processes, might help those students overcome such experiences. Furthermore, this result supports and replicates findings from recent studies that showed similar effects of school students’ (e.g., Donat et al., 2018b) and adults’ (e.g., Dzuka & Dalbert, 2007; Lipkus & Siegler, 1993) personal BJW on their offline and cyber-victimization. However, there is evidence that supports some alternative interpretations of this finding, meaning that on the one hand, massively unjust and adverse incidents of bullying might lead to a reduction of BJW (Cubela Adoric & Kvartuc, 2007). On the other hand, a strong BJW could enable students to develop strong protective factors and low risk factors to prevent or avoid being cyber-bullied. More specifically, high levels of confidence in their own future as well as an internal locus of control seem to protect students against victimization (e.g., Katz et al., 2001). In contrast, high levels of neuroticism and anxiety as well as low levels of self-concept and self-esteem were connected with a high risk of students’ victimization (e.g., Cook et al., 2010). In turn, these factors were associated with personal BJW (e.g., Dette et al., 2004; Donat et al., 2016, 2018a; Hafer, 2000; Kahileh et al., 2013). Such a development might create psychological conditions under which students can avoid becoming cyber-bullying victims in the first place (e.g., Donat et al., 2020).

Overall, it should be noted that the relations of cyber-bullying to BJW were only significant for personal but not general BJW. Thus, our results strongly support previous research in which authors stressed the necessity to distinguish between personal and general BJW (e.g., Dalbert, 1999), but also take both into account in order to investigate their unique contribution to psychological outcomes (e.g., Hafer et al., 2020). Regarding cyber-bullying among university students, personal BJW seems to be more crucial than general BJW because cyber-bullying experiences affect students’ own lives and close social environment rather than the world’s justice in general.

In line with our third hypothesis, results showed university students’ personal BJW to positively relate to their academic justice experiences. More specifically, the stronger students’ personal BJW was, the more they felt treated justly by their lecturers and fellow students. This result was non-significant for general BJW. It indicates that in particular the personal BJW functions as a psychological resource which helps university students cope with unjust experiences in the academic context via assimilation processes, for example by minimizing or denying injustice. University students with a strong personal BJW are also likely to put trust in the just behavior of their lecturers and fellow students. This finding further replicates results of recent studies that showed the relation between personal BJW and justice experiences of school students (e.g., Donat et al., 2020) and university students (e.g., Münscher et al., 2020).

The relations of lecturer and fellow student justice to cyber-bullying perpetration and victimization were of additional interest to our study. The results showed, as expected in our fourth and fifth hypothesis, that the more university students felt justly treated by their lecturers or fellow students, the less likely they were to self-report cyber-bullying perpetration and victimization. These findings are in line with previous studies on school-students’ justice experiences and involvement in offline and cyber-bullying (e.g., Donat et al., 2018a, 2020). Just treatment is a signal to students that they are valued members in their social environment and thus socially included there (e.g., Umlauft & Dalbert, 2017). Feelings of belonging to a social group strengthen these students’ individual obligation to act in conformance with this group’s rules and make cyber-bullying (Wong et al., 2014) and also other kinds of rule-breaking behavior unlikely (Emler & Reicher, 2005). This is important for the avoidance of cyber-bullying perpetration but also decreases the probability of victimization in a social environment where students are esteemed, included, and appendant. Furthermore, rule acceptance and observance are likely to be generalized to other social domains such as the judiciary or the law (Gouveia-Pereira et al., 2003; Sanches et al., 2012). Thus, justice experiences play an especially important role in explaining cyber-bullying perpetration, which also often occurs outside the academic context (e.g., Kowalski et al., 2014). Still, our research indicates that lecturers and fellow students are significant sources of such experiences and consequently associated with the occurrence of cyber-bullying.

In our sixth hypothesis, we expected university students’ affective or cognitive empathy to be negatively related to cyber-bullying perpetration. In line with this, results showed that the more the students endorsed affective or cognitive empathy, the less likely they self-reported cyber-bullying perpetration. This result supports recent research in which empathy was considered a fundamental resource of people when they interact and communicate in their social environment (Del Rey et al., 2016). Empathy may help people in general and university students in particular avoid aggressive behavior and seems to be essential for prosocial acts (Jolliffe & Farrington, 2004). Students with a strong endorsement of empathy are able to understand and share cyber-victimized people’s suffering and consequently avoid acting aggressively themselves. Studies on the relation between affective or cognitive empathy and cyber-bullying perpetration among university students showed mixed results and indicated the special role of affective empathy for cyber-bullying (e.g., Kokkinos et al., 2014). In contrast, both empathy dimensions negatively related to cyber-bullying perpetration in our study; the corresponding effects were comparably strong. Thus, university students were likely to profit from affectively sharing and cognitively understanding victimized people’s suffering in order to avoid online perpetration. In future studies, researchers should therefore continue to differentiate between both empathy dimensions when investigating cyber-bullying and other forms of online and/or aggressive behavior.

The statistical analyses also involved a control for confounding effects of student gender, internet use, and social desirability. Male students were more likely than females to self-report cyber-bullying perpetration and victimization, which is in line with the findings of some previous studies (e.g., see Kokkinos et al., 2014, for a review; Zalaquett & Chatters, 2014), but also contradicts other studies that indicated a higher risk for women to be cyber-bullied (e.g., Kokkinos et al., 2014; Larrañaga et al., 2016). Additionally, in line with Zalaquett and Chatters’s (2014) study, average daily internet use was unrelated to cyber-bullying perpetration and victimization. Furthermore, it seems to be important to control for effects of university students’ tendency to social desirability responding because it was negatively connected with their self-reports of cyber-bullying perpetration and victimization. This result supports previous research showing that this tendency was especially important when people were asked to honestly unfold sensitive personal information such as confessing deviant behavior or victimization (e.g., Bell & Naugle, 2007).

## Limitations

There are some limitations to our research that should be noted. Our data are cross-sectional, which means that causal conclusions cannot be drawn. Thus, a longitudinal study would be necessary to examine how university students’ cyber-bullying perpetration and victimization will change over time and how they will be affected by justice beliefs and experiences. Following on from this, we refused to test mediation effects of students’ academic justice experiences regarding their lecturers and fellow students on the relation between BJW and cyber-bullying although such mediation might be plausible from a theoretical or even empirical perspective (e.g., Donat et al., 2018a, 2020). Researchers have emphasized the necessity to use longitudinal data in order to test mediation effects because “cross-sectional data implicitly undermines [sic] an assumption of the statistical mediation model” (Fairchild & McDaniel, 2017, p. 1265) and “cross-sectional examination of mediation will typically generate biased estimates” (Maxwell & Cole, 2007, p. 39). Future studies should consider investigating mediation effects in a longitudinal research design.

Additionally, using self-report measures can lead to overestimations of common variance. Furthermore, data of the second sample were collected during the first months of the SARS-CoV-2 pandemic by using an online questionnaire, whereas data of the first sample were collected before the pandemic by using a paper-pencil questionnaire. Thus, these conditions might have caused systematic differences in our results between both samples because, for example, students in the second sample were likely to have used the internet more often and more intensively due to online courses at their university than students in the first sample. However, we checked for such differences and showed measurement invariance, which means that such differences are unlikely.

Another limitation is that our sample was not representative because, for example, the majority of our participants were female university students and all were German. Thus, the current findings are generalizable to other contexts or countries only to a limited extent. As a consequence, future research should focus on substantiating and replicating these results in other countries.

As discussed above, we considered some potentially confounding factors yet further characteristics might also be connected with cyber-bullying such as university students’ motives for engaging in cyber-bullying. These motives might be based on the revenge for experiences of offline victimization in the distant past or even at school, self-empowerment, demonstration of technological skills or superiority, fun, or boredom (e.g., Larrañaga et al., 2016; Vandebosch & van Cleemput, 2008). Additionally, cyber-bullying-behavior might also be associated with personality traits besides BJW and empathy, such as social intelligence, and narcissism (Kowalski et al., 2014). Moreover, there might also be justice experiences of important other people in the social environment of university students not considered here, for example friends or family. The consideration of these factors together with those studied here can help researchers draw a more complete picture of the psychological processes underlying university students’ cyber-bullying perpetration or victimization and consequently develop effective methods for cyber-bullying prevention.

Although the measure of students’ cyber-bullying experiences differentiates several forms of such behavior (e.g., outing, flaming, threat), we refused to relate BJW, empathy, or justice experiences to each of these forms separately. In future studies, the significance of different bullying forms for these relations could be examined. Our results were quite similar to those for cyber-bullying in school students and offline bullying (e.g., Donat et al., 2018a, b, 2020), which reflects a strong correspondence between the phenomena (e.g., Kowalski et al., 2014). However, it is still not clear how the investigated relations would vary depending on different types of offline or cyber-bullying, especially across different life spans (e.g., adolescence, young adulthood).

## Conclusion

An important conclusion from our study is that not only university students’ personal BJW and empathy but also their academic justice experiences related to cyber-bullying perpetration or victimization. Thus, researchers should develop strategies in order prevent or reduce cyber-bullying, which (1) should focus on simultaneously fostering their personal BJW and empathy, (2) consider especially the behavior of lecturers and fellow students, who are an important source of justice experiences, (3) thereby apply aspects of interactional justice (e.g., Peter et al., 2013), such as treating each other with respect, civility, and dignity, (4) take the subjective nature of justice experiences into account (e.g., Dalbert et al., 2007), and altogether establish an academic environment that allows students and lecturers to develop or enhance a strong personal BJW and a strong tendency to understand and share other people’s feelings. This might contribute to a just social climate at university and the development of appropriate social-emotional competences of young adults there. Due to the novelty of our approach, we still need to identify the specific behavior—particularly in association with cyber-bullying—which university students judge as more or less just.

## Supplementary Information

ESM 1 (DOCX 51 kb)

## Data Availability

The datasets generated during and/or analysed during the current study are available in the PsychArchives repository, 10.23668/psycharchives.5597
