# Supplementary material for: Cyber-bullying among university students: Concurrent relations to belief in a just world and to empathy
Source: Curr Psychol. 2022 Jun 1;42(10):7883–96. doi: 10.1007/s12144-022-03239-z (PMC9155235; doi:10.1007/s12144-022-03239-z)
Supplement: Supplementary file 1 — (DOCX 51 kb) [file 12144_2022_3239_MOESM1_ESM.docx]

**Supplement 1**

*Supplement 1.1*

*Summary of Measurement-Invariance Test by the R-Package lavaan* (Rosseel et al., 2018)

| Model | *df* | *df* difference | Χ^2^ | Χ^2^ difference | *p* |
| --- | --- | --- | --- | --- | --- |
| 1 fit.configural | 7544 | - | 7123.80 | - | - |
| 2 fit.loadings | 7622 | 78 | 7915.40 | 5.76 | 1.00 |
| 3 fit.intercepts | 7700 | 78 | 7967.20 | 7.63 | 1.00 |
| 4 fit.means | 7711 | 11 | 8105.20 | 3.28 | 0.99 |

*Note*. Scaled Chi-Squared Difference Test (method = “satorra.bentler.2001”, Rosseel et al., 2018).

*Supplement 1.2*

*Fit Measures of the Measurement Invariant Test*

| Model | CFI scaled | RMSEA scaled | ΔCFI scaled | ΔRMSEA scaled |
| --- | --- | --- | --- | --- |
| 1 fit.configural | .833 | .012 | - | - |
| 2 fit.loadings | .839 | .011 | .006 | < .001 |
| 3 fit.intercepts | .838 | .011 | .001 | < .001 |
| 4 fit.means | .833 | .011 | .006 | < .001 |

*Note*. Scaled Chi-Squared Difference Test (method = “satorra.bentler.2001”, Rosseel et al., 2018).

**Supplement 2**

*Latent Correlations and Regression Weights from a Latent Structural Equation Model (N = 615 German University Students), Additional Results*

| Variables | *Corr*_(lat)_ | *SE* | *z* | *p* | CI_95%_ | |
| --- | --- | --- | --- | --- | --- | --- |
| Affective empathy ~~ |  |  |  |  |  |  |
| Personal BJW | .12 | 0.06 | 2.06 | .04 | .01 | .24 |
| General BJW | < .01 | 0.06 | -0.01 | .99 | -.12 | .12 |
| Lecturer justice | .18 | 0.06 | 3.32 | < .01 | .08 | .29 |
| Fellow student justice | .15 | 0.05 | 2.69 | .01 | .04 | .25 |
| Social desirability | .53 | 0.05 | 9.92 | < .01 | .42 | .63 |
| Cognitive empathy ~~ |  |  |  |  |  |  |
| Affective empathy | .63 | 0.05 | 13.71 | < .01 | .54 | .72 |
| Personal BJW | .18 | 0.06 | 3.07 | < .01 | .07 | .30 |
| General BJW | < .01 | 0.06 | -0.03 | .98 | -.11 | .11 |
| Lecturer justice | .18 | 0.06 | 3.23 | < .01 | .07 | .28 |
| Fellow student justice | .19 | 0.05 | 3.65 | < .01 | .09 | .30 |
| Social desirability | .62 | 0.05 | 13.29 | < .01 | .53 | .71 |
| Cyber-bullying perpetration ~~ |  |  |  |  |  |  |
| Cyber-bullying victimization | .87 | 0.05 | 17.65 | < .01 | .77 | .96 |
| Person BJW ~~ |  |  |  |  |  |  |
| General BJW | .41 | 0.04 | 9.37 | < .01 | .33 | .50 |
| Social desirability | .37 | 0.05 | 7.14 | < .01 | .27 | .47 |
| General BJW ~~ |  |  |  |  |  |  |
| Social desirability | .23 | 0.06 | 4.09 | < .01 | .12 | .34 |
| Lecturer justice ~~ |  |  |  |  |  |  |
| Fellow student justice | .71 | 0.04 | 16.15 | < .01 | .63 | .80 |
| Social desirability | .25 | 0.06 | 4.21 | < .01 | .13 | .37 |
| Fellow student justice ~~ |  |  |  |  |  |  |
| Social desirability | .31 | 0.06 | 5.67 | < .01 | .20 | .42 |
| Variables | β | *SE* | *z* | *p* | CI_95%_ | |
| Affective empathy ~ |  |  |  |  |  |  |
| Gender | -.33 | 0.05 | -7.00 | < .01 | -.42 | -.24 |
| Internet use | < .01 | 0.05 | 0.00 | 1.00 | -.09 | .09 |
| Cognitive empathy ~ |  |  |  |  |  |  |
| Gender | -.08 | 0.05 | -1.64 | .10 | -.17 | .02 |
| Internet use | .04 | 0.05 | 0.80 | .42 | -.06 | .14 |
| Personal BJW ~ |  |  |  |  |  |  |
| Gender | -.03 | 0.05 | -0.67 | .50 | -.13 | .07 |
| Internet use | .01 | 0.04 | 0.23 | .82 | -.07 | .09 |
| General BJW ~ |  |  |  |  |  |  |
| Gender | -.04 | 0.05 | -0.83 | .41 | -.14 | .06 |
| Internet use | -.01 | 0.05 | -0.29 | .78 | -.11 | .08 |
| Lecturer justice ~ |  |  |  |  |  |  |
| Gender | -.05 | 0.05 | -1.04 | .30 | -.14 | .04 |
| Internet use | .07 | 0.04 | 1.91 | .06 | .00 | .15 |
| Fellow student justice ~ |  |  |  |  |  |  |
| Gender | -.09 | 0.05 | -1.76 | .08 | -.19 | .01 |
| Internet use | .01 | 0.04 | 0.21 | .84 | -.08 | .09 |
| Social desirability ~ |  |  |  |  |  |  |
| Gender | -.22 | 0.05 | -4.23 | < .01 | -.33 | -.12 |
| Internet use | .04 | 0.05 | 0.80 | .42 | -.06 | .14 |

*Note*. *Corr*_(lat)_ = latent correlation. BJW = belief in a just world. Cyber-bullying perpetration and victimization ranged between 1 and 5, all other continuous variables between 1 and 6, with higher values indicating a stronger endorsement of the constructs. For gender, 1 = female, 2 = male. Internet use: average hours spent daily on the internet.
